# Antibiotic use among children under five years with diarrhea in rural communities of Gulu, northern Uganda: a cross-sectional study

**DOI:** 10.1186/s12889-021-11254-1

**Published:** 2021-06-29

**Authors:** Hindum Lanyero, Moses Ocan, Celestino Obua, Cecilia Stålsby Lundborg, Sarah Nanzigu, Agaba Katureebe, Joan N. Kalyango, Jaran Eriksen

**Affiliations:** 1 Department of Pharmacology and Therapeutics, Makerere University College of Health Sciences, Kampala, Uganda; 2 Mbarara University of Science and Technology, Mbarara, Uganda; 3 Department of Global Public Health, Karolinska Institutet, Tomtebodavägen 18 A, 171 77 Stockholm, Sweden; 4 Infectious Diseases Research Collaboration, Kampala, Uganda; 5 Department of Pharmacy, Makerere University College of Health Sciences, Kampala, Uganda; 6 Clinical Epidemiology Unit, Makerere University College of Health Sciences, Kampala, Uganda; 7 Department of infectious diseases, South General Hospital, 118 83 Stockholm, Sweden

## Abstract

**Background:**

Diarrhea is the second leading cause of mortality in children under 5 years of age globally, and the risk of death increases with practices such as restriction of fluid intake and inappropriate use of antibiotics. We determined the prevalence of antibiotic use in managing diarrhea in children under 5 years of age in rural communities of Gulu district, northern Uganda.

**Method:**

A cross-sectional study among children under 5 years with diarrhea, from households selected using multi-stage sampling. A researcher administered questionnaire was used to obtain data from caregivers of these children.

**Results:**

Of the 856 children recruited, 318 (37.1%, 318/856) had experienced diarrhea, where 263 (82.7%, 263/318) had diarrhea with acute respiratory infections (ARIs), and 55 (17.3%, 55/318) had diarrhea without ARIs. The majority (89.6%, 285/318) of the children had non-bloody diarrhea. A high proportion (82.8%) of the children with non-bloody diarrhea also had ARIs. Bloody diarrhea was reported for 33 (10.4%) children including those with ARIs, and only 6 of these (18.2%) children had bloody diarrhea without ARIs. Of the 318 children with diarrhea, over half (52%, CI: 46–57) were administered antibiotics. Of the 55 children who had diarrhea without ARIs, over a third (38%, CI: 26–51) were administered antibiotics. Similarly, of the 263 children with diarrhea and ARIs, 54% (CI: 48–60) were treated with antibiotics. The determinants of antibiotic use included; children living in peri-urban settings (AOR: 3.41, CI: 1.65–7.08, *P* = 0.001), getting treatment from health facility (AOR: 1.76, CI: 1.06–2.93, *P* = 0.029), and having diarrhea with ARIs (AOR: 3.09, CI: 1.49–6.42, *P* = 0.003).

**Conclusion:**

Antibiotic use is common among children under 5 years with diarrhea in rural communities of northern Uganda.

## Background

Diarrhea is one of the leading causes of morbidity and mortality in children under 5 years globally, with about 1.7 billion episodes and 578,000 deaths every year [1–3]. Most of these episodes and deaths occur among children in Africa with about 440 million cases and 350,000 deaths annually [3, 4]. In Uganda, the prevalence of diarrhea in children under 5 years is 23%, with an average of 3.2 episodes of diarrhea per child per year resulting in about 6857 deaths among children under 5 years per year in Uganda [4, 5].

Infectious diarrhea is the most common type of diarrhea among children under 5 years in low and middle income countries (LMICs), caused by a variety of bacterial, viral and parasitic pathogenic organisms [6]. Infection is spread through contaminated food or drinking-water, or from person-to-person as a result of poor hygiene [1]. In Uganda, poor sanitation and hygiene, as well as unequal access to safe drinking water, exposes thousands of children to diarrhea. Nearly a tenth of the population has been reported to practice open defecation, and two thirds of households do not wash hands with soap [7]. Higher mortality rates in children with diarrhea is attributed to risk factors such as younger age, male gender, early weaning, seasonal patterns, low maternal education, poor water storage, lack of hand washing with soap and inappropriate treatment [2].

The WHO guidelines recommend use of oral rehydration solution (or an intravenous electrolyte solution in cases of severe dehydration) as well as zinc supplementation and continued breast feeding as treatment for diarrhea [8], while antibiotics are recommended in cases of bloody diarrhea, suspected cholera, severe malnutrition or associated sepsis [8]. However, appropriate treatment of diarrhea still remains a problem in many LMICs [1, 8]. Studies have reported a high degree of inappropriate treatment practices such as the indiscriminate use of antibiotics [3, 9–15]. A meta-analysis by Auta et al. reported a high prevalence (23.1%) of antibiotic use in children under 5 years with non-bloody diarrhea in Sub-Saharan Africa [6]. Another study that assessed the management and treatment practices for acute pediatric diarrhea in Nigeria, reported a prevalence of 85% antibiotic use in the treatment of diarrhea [9]. In a survey to assess practices of antibiotic prescription at registered drug shops in Uganda, 29.4% of the drug shop providers interviewed reported that antibiotics were the first-line treatment for children with diarrhea and yet the standard guideline is to give oral rehydration salts and zinc tablets [16]. According to another study in Uganda which explored the knowledge and practices of diarrhea case management among health care providers at health centers and drug shops; it was reported that 81% of staff in health centers and 87% of those in drug shops prescribed antibiotics for common diarrhea [17]. Finally, in a survey to determine the factors associated with the use of antibiotics in the management of non-bloody diarrhea in children under 5 years of age in sub-Saharan Africa, it was reported that, the use of antibiotics was associated with the source of care, place of residence, wealth index, maternal education and breastfeeding status [3].

Inappropriate antibiotic use in diarrhea is a potential driver of resistance development [3, 18] and could worsen the course of disease especially when broad spectrum agents are used due to their effects on the gut microflora [19].

The main drivers of antibacterial misuse in treatment of diarrhea in children include, self-medication, inadequate healthcare infrastructure, limited therapeutic options due to widespread resistance and poor prescription practices [3, 18].

Due to the more than two decades of war in the region, the healthcare infrastructure was affected and is currently recovering at a slow pace thus lagging behind the rest of the country in service delivery especially in the rural settings [20]. Previous studies in LMICs have reported antibiotic misuse in management of diarrhea in children under 5 years [3, 9–14, 16, 17, 21], however, these reports may not be representative of the extent of antibiotic use in children under 5 years with diarrhea in post conflict areas in LMICs. Therefore, there was need to carry out this study to help provide evidence on the extent of antibiotic use in children under 5 years with diarrhea in Gulu, northern Uganda. The information generated from this study if considered by policy makers could be a useful guide in designing interventions to mitigate inappropriate antibiotic use in diarrhea, hence reduce the risk of emergence of antibiotic resistance in the community.

## Methods

### Study design, site and population

This was a cross-sectional survey carried out in households in rural communities in Gulu district, northern Uganda between November, 2018 and February 2019. Gulu district is over 300 km north of the capital city of Uganda, Kampala. Data on antibiotic use in children under 5 years with diarrhea were collected from the childrens’ caregivers. According to the national population census in 2014, the population of children under 5 years in the area was estimated to be 43,067 [22].

### Sample size determination

We calculated the number of children needed using a formula for estimation of a single proportion with adjustment of sample size to cater for multiple stage cluster sampling [23]. We assumed; the proportion of children getting antibiotic treatment for diarrhea and/or Acute respiratory infections (ARIs) to be 50%, a 95% level of confidence, power of 80% sample design effect of 2.0, and 10% adjustment for non-response. The sample size of 865 was obtained.

### Sampling criteria

**Fig. 1:**
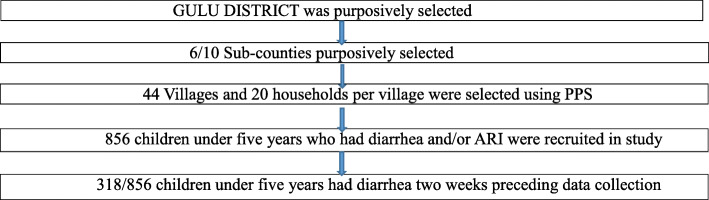
Multi-stage sampling

Gulu district in northern Uganda having been the epicenter of the war was purposively selected for this study and multi-stage sampling used in selecting the study units (Fig. 1). Gulu district is administratively divided into ten (10) sub-counties. A total of six (6) sub-counties were purposively selected to represent high and low population areas [24].

In each of the sub-counties, probability proportionate to size (PPS) was used in estimating the number of villages per sub-county and number of households per village to be sampled [25]. Forty-four (44) villages were selected and in each village 20 households were selected using PPS formula. One child that had symptoms of diarrhea and/or ARIs 2 weeks preceding data collection day was selected from each household for inclusion into the study. In the event that a household had more than one child under 5 years who had suffered from diarrhea and/or ARIs 2 weeks prior to data collection date, one child was selected using simple random sampling. For the purpose of this study, we defined diarrhea as the passage of three or more loose or liquid stools per day (or more frequent passage than is normal for the individual) [1].

### Data collection tool

Data on antibiotic use in the treatment of diarrhea in children under 5 years were collected using a structured interviewer administered questionnaire. The questionnaire was developed specifically for this study and has been published in another paper which assessed the use of antibacterials in the management of symptoms of acute respiratory tract infections among children under 5 years in Gulu, northern Uganda: Prevalence and determinants [24]. The tool was pre-tested in 60 households and modified to capture all variables of interest and any question which was not useful was removed.

### Data collection

Written informed consent was sought from care-takers of children under 5 years with diarrhea and/or ARIs and those who consented were interviewed using an interviewer administered questionnaire. Final year pharmacy students at Gulu University were trained on the data collection tool and collected field data. Information was collected on i) demographic characteristics, ii) Illness characteristics by establishing whether the child had loose stool with blood (bloody diarrhea), loose stool without blood (non-bloody diarrhea), and having ARIs, and iii) Information on medicine use in management of diarrhea in children under 5 years. The interview lasted about 30 min.

### Data management

Data collection tools were checked for completeness at the end of each field data collection day by the principal investigator (HL) and any inconsistence addressed by discussing with the research assistants.

Double data entry was done in Epi-Data 3.1 software. The two datasets were then cleaned and analyzed in STATA 14.0.

### Statistical analysis

Means, standard deviation (SD) and frequency were used to describe the characteristics of the study participants. Clustered robust standard error was used to compute the factor specific prevalence of antibacterial use with village as the clustering variable. Random effects logistics regression was used to determine the factors associated with antibiotic use. In the univariable analysis each independent variable was regressed with the dependent variable adjusting for clustering variable, village to determine the crude associations. All factors which achieved a *p*-value of less than 0.1 were considered fit to be included in the multivariable model. The model was built using backward elimination algorithm and statistical significance of the explanatory variables were assessed using likelihood ratio test. Appropriateness of the model was tested using the Hosmer-Lemeshow test. All statistical inferential frame works were based on the two-sided *P*-value and a 5% error margin.

## Results

A total of 856 children under 5 years were recruited in the study based on the inclusion criteria, which was having diarrhea and/or ARIs. In this paper we report on children who had diarrhea, another paper on ARIs has been published [24].

### Socio-demographic and illness characteristics of the caregivers and children under 5 years

Of the 856 children recruited in the study, 318 children had diarrhea or had had diarrhea during the 2 weeks preceding our field visit. Over half of the children with diarrhea (55%, *n* = 175/318) were male. The average age of children under 5 years was 19.5 ± 4.8 (SD) months. The caregivers were mainly female (97.8%, *n* = 311/318), and the majority (90%, *n* = 286/318) of the caregivers were mothers to the children. The caregivers were mostly farmers (58.8%, *n* = 187/318). The majority (68.9%, *n* = 219/318) of the caregivers and their children lived within 1-h walking distance to the nearest health facility and in rural areas (82.1%, *n* = 261/318). Distance was measured in walking distance as this was the most common way to get to the health facilities.

Of the 318 children who had diarrhea in this study, 285 (89.6%, 285/318) had non-bloody diarrhea, 33 (10.4%, 33/318) had bloody diarrhea, 236 (74.2%, 236/318) had non bloody diarrhea with ARIs, and 49 (15.4%, 49/318) had non-bloody diarrhea without ARIs (Table 1).

**Table 1:** Socio-demographic and illness characteristics of child caregivers and children under 5 years

Characteristics	Categories	*N* = 318 Measure, n (%)
Sex of child, n (%)	Male	175 (55.0)
	Female	143 (45.0)
Location of household, n (%)	Rural	261 (82.1)
	Peri-urban	57 (17.9)
Sex of child caregiver, n (%)	Female	311 (97.8)
	Male	7 (2.2)
Education of caregiver	No education	178 (56.0)
	Primary	90 (28.3)
	O Level	27 (8.5)
	A Level	5 (1.6)
	Tertiary	18 (5.7)
Occupation of caregiver	Peasant	187 (58.8)
	Skilled Labor	20 (6.3)
	Business Owner	77 (24.2)
	Unskilled Labor	34 (10.7)
Distance to health facility (in travel time)	Within 1 Hour	219 (68.9)
	Within 2 Hours	65 (20.4)
	≥ 3 Hours	34 (10.7)
Diarrhea	Bloody diarrhea	33 (10.4)
	Non bloody diarrhea	285 (89.6)
Diarrhea with ARIs	No	55 (17.3)
	Yes	263 (82.7)
Non-bloody diarrhea without ARIs	No	269 (84.6)
	Yes	49 (15.4)
Non-bloody diarrhea with ARIs	No	82 (25.8)
	yes	236 (74.2)
Bloody diarrhea without ARIs	No	312 (98.1)
	yes	6 (1.9)
Age of child (months), mean (SD)		19 (4.8)
Age of child caregiver (years), mean (SD)		27 (8.4)

*n* sample size, *SD* Standard Deviation, *%* percentage

### Prevalence of antibiotic use in children under 5 years with diarrhea in northern Uganda

Out of 318 children under 5 years who had diarrhea (with or without ARIs), 164 (52%; CI: 46–57) were treated with antibiotics. Among children, the prevalence of antibiotic use was higher in males (53%, CI: 45–60), those in the age group of 37–59 months (56%, CI: 39–72), those from peri-urban areas (65%, CI: 52–76) and in those who got treatment from a health facility (53%, CI: 45–60). The prevalence of antibiotic use was also higher in children whose caregivers were male (71%, CI: 30–94), had O-level education (63%, CI: 43–79), were a skilled laborer (70%, CI: 47–86), and in the age group ≥45 years (62%, CI: 40–80).

Among the 285 children who had non bloody diarrhea, 142 (50%; CI: 44–56) were treated with antibiotics. Similarly, of the 236 children who had non-bloody diarrhea with ARIs, 127 (54%; 47–60) were treated with antibiotics and of the 49 children who had only non-bloody diarrhea (without having ARIs) 19 (39%; CI: 26–53) were treated with antibiotics (Table 2).

**Table 2:** Prevalence of antibiotic use in children under 5 years with diarrhea

Variable	Description	Proportion of antibacterial use, n (%)	95% CI
Overall		164 (52)	46–57
Age of child (months)	1–12	60 (53)	43–62
	13–36	85 (50)	42–58
	37–59	19 (56)	39–72
Sex of child	Male	92 (53)	45–60
	Female	72 (50)	42–59
Location of household	Rural	127 (49)	43–55
	Peri-urban	37 (65)	52–76
Education of caregiver	No education	86 (48)	41–56
	Primary	48 (53)	43–63
	O Level	17 (63)	43–79
	A Level	3 (60)	17–92
	Tertiary	10 (56)	32–77
Age caregiver	15–24	73 (47)	39–55
	25–34	62 (56)	46–65
	35–44	16 (53)	35–70
	≥ 45	13 (62)	40–80
Sex of caregiver	Male	5 (71)	30–94
	Female	159 (50)	44–55
Occupation of caregiver	Peasant	84 (45)	38–52
	Skilled Labor	14 (70)	47–86
	Business Owner	44 (57)	46–68
	Unskilled Labor	22 (65)	47–79
Distance to health facility (in travel time)	Within 1 Hour	116 (50)	44–57
	Within 2 Hours	33 (51)	39–63
	≥ 3 Hours	15 (47)	31–64
Health facility use	No	41 (48)	37–58
	yes	123 (53)	47–59
Diarrhea	Bloody diarrhea	17 (52)	34–69
	Non-bloody diarrhea	142 (50)	44–56
Diarrhea with ARIs	No	21 (38)	26–51
	Yes	143 (54)	48–60
Non-bloody diarrhea without ARIs	No	145 (54)	48–60
	Yes	19 (39)	26–53
Non-bloody diarrhea with ARIs	No	37 (45)	34–57
	Yes	127 (54)	47–60
Bloody diarrhea without ARIs	No	162 (52)	46–57
	yes	2 (33)	7–76

CI: Confidence Interval; n: sample size; %: percentage

### Factors associated with antibiotic use in children under 5 years with diarrhea

In the univariable analysis, the following factors were found to have a *P*-value of less than 0.1 and were used to build the multivariable model: location of household (children from peri-urban areas, *P* = 0.034), occupation of caregiver (caregiver being a skilled laborer, *P* = 0.04, unskilled laborer *P* = 0.038), health facility use (getting treatment from a health facility, *P* < 0.001), having diarrhea without ARIs (*P* = 0.031), having diarrhea with ARIs (*P* = 0.031) and having non-bloody diarrhea (*P* = 0.051). Having bloody diarrhea without ARIs, age of child and sex of child were included in the model as a prior since studies have shown that they are significantly associated with antibiotic use in children with diarrhea [3, 8] (Table 3).

**Table 3:** Factors associated with antibiotic use in children under 5 years with diarrhea

Variable	Description	COR (95% CI)	*P*-value
Age of child (months)	1–12	1.00	
	13–36	0.90 (0.55–1.46)	0.670
	37–59	1.13 (0.51–2.48)	0.762
Sex of child	Male	1.00	
	Female	0.91 (0.58–1.43)	0.692
Location of household	Rural	1.00	
	Peri-urban	1.98 (1.05–3.71)	0.034
Education of caregiver	No education	1.00	
	Primary	1.19 (0.70–2.02)	0.510
	O Level	1.78 (0.76–4.18)	0.187
	A Level	1.55 (0.24–9.87)	0.644
	Tertiary	1.31 (0.48–3.55)	0.598
Age caregiver	15–24	1.00	
	25–34	1.46 (0.88–2.42)	0.143
	35–44	1.36 (0.61–3.05)	0.456
	≥ 45	1.92 (0.73–5.05)	0.186
Sex of caregiver	Male	1.00	
	Female	0.40 (0.07–2.19)	0.293
Occupation of caregiver	Peasant	1.00	
	Skilled Labor	2.86 (1.05–7.79)	0.040
	Business Owner	1.63 (0.95–2.80)	0.077
	Unskilled Labor	2.25 (1.05–4.84)	0.038
Time to reach health facility	Within 1 Hour	1.00	
	Within 2 Hours	0.92 (0.51–1.64)	0.775
	≥ 3 Hours	0.66 (0.31–1.42)	0.287
Health facility use	No	1.00	
	yes	38.94 (15.19–99.79)	< 0.001
Diarrhea with ARIs	No	1.00	
	Yes	1.98 (1.06–3.69)	0.031
Diarrhea without ARIs	No	1.00	
	Yes	0.50 (0.27–0.93)	0.031
Non-bloody diarrhea without ARIs	No	1.00	
	Yes	0.52 (0.27–1.00)	0.051
Bloody diarrhea without ARIs	No	1.00	
	yes	0.49 (0.08–2.83)	0.426

*COR* Crude Odds Ratio, *CI* Confidence Interval

In the multivariable model, children in peri-urban settings were three times more likely to be given antibiotics compared to those in rural settings (AOR: 3.41, CI: 1.65–7.08). Children who were taken to a health facility were more likely to use antibiotics compared to those who were not (AOR: 1.76, CI: 1.06–2.93). Children who had diarrhea with ARIs had three times the odds to be given antibiotics compared to those who had diarrhea without ARIs (AOR: 3.09, CI: 1.49–6.42) (Table 4).

**Table 4:** Multivariable analysis of factors associated with antibiotic use in children under 5 years

Variable	Description	AOR (95% CI)	*P*-value
Child Age (months)	1–12	1.00	
	13–36	0.66 (0.39–1.11)	0.119
	37–59	1.53 (0.65–3.60)	0.333
Sex of child	Male	1.00	
	Female	0.92 (0.57–1.48)	0.721
Location of household	Rural	1.00	
	Peri-urban	3.41 (1.65–7.08)	0.001
Age caregiver (years)	15–24	1.00	
	25–34	1.75 (1.02–2.98)	0.041
	34–44	1.32 (0.57–3.09)	0.518
	≥ 45	1.72 (0.62–4.79)	0.300
Diarrhea with ARIs	No	1.00	
	Yes	3.09 (1.49–6.42)	0.003
Bloody diarrhea without ARIs	No	1.00	
	Yes	0.94 (0.42–2.12)	0.886
Treatment at health facility	No	1.00	
	Yes	1.76 (1.06–2.93)	0.029

*AOR* Adjusted odds ratio, *%* Percentage, *CI* Confidence Interval

## Discussion

In this study we demonstrated that the prevalence of antibiotics use in children under 5 years with diarrhea was high. Over half (52%) of the children with diarrhea and 50% of those with non-bloody diarrhea were treated with antibiotics. We also observed that, 39% of the children who had non- bloody diarrhea without ARIs and 54% of those who had non-bloody diarrhea with ARIs were treated with antibiotics. Getting treatment from a health facility, children from peri-urban areas, and children having diarrhea with ARIs were significantly associated with antibiotic use in children under 5 years with diarrhea.

Antibiotics are usually not recommended in children with diarrhea except in cases of bloody diarrhea, cholera, severe malnutrition, or when the diarrhea is associated with sepsis. However, in this study we report an overuse of antibiotics in children under 5 years with non-bloody diarrhea. The overuse of antibiotics in children under 5 years with non-bloody diarrhea reported in this study is similar to those from previous studies in low and middle income settings [11, 12, 26]. A study in Kashmir, India reported an even higher (77.9%) rate of antibiotic use in managing children under 5 years with diarrhea [27]. In this study, and the one from Kashmiri, it was observed that some of the children did not only have diarrhea but they also had other concurrent symptoms such as those of ARIs [27]. It is therefore possible that some of these children could have received these antibiotics for treatment of other extraintestinal infections. In this study we observed that children who had non-bloody diarrhea without ARIs had a lower prevalence (39%) of antibiotics use compared to those who had non-bloody diarrhea with symptoms of ARIs (54%). Similarily, another study from Kenya, also reported a high prevalence of antibacterial use among children who had diarrhea with ARIs compared to those who only had diarrhea [28]. In Uganda, antibiotics are prescription only medicines [29], however, there is inadequate enforcement of regulation of access to antibiotics, making them easily available and possible to self-medicate [30]. This, coupled with non-functional microbiology laboratories in most healthcare facilities could be responsible for the overuse of antibiotics in children with diarrhea observed in this study. The overuse of antibiotics is a potential driver of antibacterial resistance emergency and spread [31], making previously treatable infections fatal and increasing costs for health-care and society. A study was carried out in Uganda to determine the epidemiology and antibiotic susceptibility of Vibrio cholerae associated with the 2017 outbreak in Kasese district, and it reported that *V. cholerae* was highly resistant to the commonly used antibiotics [32].

The overuse of antibiotics was higher in the group that was treated at a health facility. This finding is similar to those from previous studies; a study in a Mexican community reported that patients who were seen by a physician were 6 times likely to be treated with an antibiotic for diarrhea compared to those who did not consult a physician [33]. Similarly, a meta-analysis of health survey data in sub-Saharan Africa reported that those children who did not seek treatment from a healthcare provider were less likely to be treated with antibiotics for diarrhea [3]. Studies have shown that the higher the level of education of a healthcare worker, the less likely they are to use antibiotics inappropriately in management of diarrhea [10], and in Uganda, we find that healthcare workers of low cadre tend to work in the rural communities, and this could explain the high prevalence of antibiotics use in rural health centers [34, 35].

In this study, children whose households were located in peri-urban areas were more likely to receive antibiotic treatment compared to those in rural areas, a finding similar to that from a meta-analysis done in 30 countries in sub-Saharan Africa, which reported a higher prevalence of antibiotic use in urban (26.7%) compared to rural (21.6%) areas (*P* < 0.001) [3]. This could be because the peri-urban population has better access to, and more information about, health care services, as compared to those in rural areas. Being highly educated and getting treatment from health facilities [3] have been reported to be associated with higher antibacterial use in LMICs (*P* < 0.001) .

According to this study, children who had diarrhea with ARIs were more likely to be given antibiotics compared to those who had diarrhea without ARIs. This finding is similar to that from a study in Kenya where children who had diarrhea with concurrent ARIs were over prescribed antibiotics compared to those who had diarrhea without ARIs [28]. It is therefore possible that some of these children could have been given antibacterials to treat ARIs and not diarrhea.

The results of our study should be considered in light of some limitations. For example, by including children with diarrhea and ARIs, we were unable to generate actionable recommendations since the antibiotics could have been given to treat the ARIs. Only 55 children had diarrhea without any co-morbidity, but despite the small number our findings show that there is inappropriate antibiotic use in children with non-bloody diarrhea. We were unable to ascertain whether some of the children had invasive non-bloody diarrhea which requires antibiotic treatment [36, 37], because some of the caregivers self-medicated and some of those who sought treatment from healthcare facilities did not have prescriptions. We used self-reports to establish antibiotic use in children, this is likely to be affected by recall bias. However, the recall period of 14 days that we used has been found to reduce the risk of this [38, 39]. In addition, the study could have also been affected by social desirability bias during field data collection. The high level of reported antibiotic use makes this less likely. We were unable to assess factors like nutritional status of the children which could have also contributed to increased antibiotic prescription rates. Some of the respondents also did not know the name of the medicines that they had given to their children, this was addressed by requesting the respondents to describe the medicine (color, how many times it was given, taste, whether capsule or tablet). Also, the respondents were requested to bring the primary or secondary packets of the medicines or the remaining medicine if still there.

## Conclusions

The prevalence of antibiotic use is high among children under 5 years with diarrhea in rural communities of Gulu district, northern Uganda. There is overuse of antibiotic in children who present with non- bloody diarrhea without any other concurrent illness. In most of these cases antibiotic would not be necessary, instead leading to adverse effects and creating unnecessary antibiotic pressure, potentially leading to development of resistance. Getting treatment from a health facility, children from peri-urban areas, and having diarrhea with ARIs. Therefore, there is need to educate both prescribers and caregivers on appropriate management of diarrhea and the consequences of inappropriate use of antibiotics in children.

## Data Availability

The datasets generated and/or analyzed during the current study are not publicly available because they contain identifying patient information but are available from the corresponding author on reasonable request.

## Abbreviations

AOR: Adjusted Odds Ratio
ARI: Acute Respiratory Infections
CI: Confidence Interval
COR: Crude Odds Ratio
CQ: Chloroquine
LMICs: Low Middle Income Countries
P: *P*-value
PPS: Probability Proportionate to Size
SD: Standard Deviation
SP: Sulphadoxine/Pyrimethamine
WHO: World Health Organization
